# Governance hurdles for expansion of low trophic mariculture production in Sweden

**DOI:** 10.1007/s13280-024-02033-4

**Published:** 2024-05-06

**Authors:** Frida Franzén, Åsa Strand, Johanna Stadmark, Ida Ingmansson, Jean-Baptiste E Thomas, Tore Söderqvist, Rajib Sinha, Fredrik Gröndahl, Linus Hasselström

**Affiliations:** 1 Tyrens AB, Folkungagatan 44, 118 86 Stockholm, Sweden; 2 IVL Svenska Miljöinstitutet/IVL Swedish Environmental Research Institute, Kristineberg 566, 451 78 Fiskebäckskil, Sweden; 3 IVL Svenska Miljöinstitutet/IVL Swedish Environmental Research Institute, Box 530 21, 400 14 Gothenburg, Sweden; 4 Department of Sustainable Development, Environmental Science and Engineering, KTH Royal Institute of Technology, Teknikringen 10B, 100 44 Stockholm, Sweden; 5 Anthesis Enveco AB, Barnhusgatan 4, 111 23 Stockholm, Sweden; 6 Holmboe & Skarp AB, Norr Källstavägen 9, 148 96 Sorunda, Sweden

**Keywords:** Aquaculture governance, Blue economy, Blue growth, Ecosystem services, Governance constrains, Low trophic mariculture (LTM)

## Abstract

The study examines the governance of low trophic species mariculture (LTM) using Sweden as a case study. LTM, involving species such as seaweeds and mollusks, offers ecosystem services and nutritious foods. Despite its potential to contribute to blue growth and Sustainable Development Goals, LTM development in the EU and OECD countries has stagnated. A framework for mapping governance elements (institutions, structures, and processes) and analyzing governance objective (effective, equitable, responsive, and robust) was combined with surveys addressed to the private entrepreneurs in the sector. Analysis reveals ineffective institutions due to lack of updated legislation and guidance, resulting in ambiguous interpretations. Governance structures include multiple decision-making bodies without a clear coordination agency. Licensing processes were lengthy and costly for the private entrepreneurs, and the outcomes were uncertain. To support Sweden’s blue bioeconomy, LTM governance requires policy integration, clearer direction, coordinated decision-making, and mechanisms for conflict resolution and learning.

**Supplementary Information:**

The online version contains supplementary material available at 10.1007/s13280-024-02033-4.

## Introduction

The capacity of marine and coastal areas to provide ecosystem services such as climate regulation, energy, recreation, and the provision of nutritious food is essential for human societies. However, anthropogenic pressures and the usage of marine resources have severely reduced this ability, emphasizing the need of finding new sustainable ways of utilizing marine resources (Diz et al. 2019). Deviating from the current pathway is critical to transfer ocean systems within a safe space to operate, particularly regarding nitrogen (N) and phosphorus (P) cycles and biodiversity (Folke et al. 2021; Richardson et al. 2023), but also for reaching several Sustainable Development Goals (SDGs): SDG 14 (Life below water), as well as SDG 2 (Zero hunger), SDG 8 (Decent work and economic growth), and SDG 12 (Responsible consumption and production).

Social and ecological systems are nested, interdependent, and difficult to predict, and thus, governance must be flexible and adaptive (Ostrom 2007). Blue growth is a concept sprung from the need to handle the interconnection between social and ecological marine systems by providing a framework for managing marine uses and marine ecosystem services jointly (Burgess et al. 2018). In 2021, the EU Commission launched the “new approach for sustainable blue economy in the EU” to better align with the European Green deal and emphasize the oceans’ role for reaching environmental targets (European Commission 2021a). However, to reach the ambitious sustainability goals for oceans, as well as to reverse the negative trends, human actions also need to both contribute to ecosystem services and accelerate the oceans’ own ability to produce ecosystem services. Businesses or activities producing both private benefits (such as commercially valuable products) and public benefits (such as eutrophication mitigation) are referred to as genuine blue growth (Hasselström and Gröndahl 2021).

Aquaculture, the farming of aquatic plants and animals, is one of the targeted sectors for blue growth (European Commission 2021b) and shows great promise for genuine blue growth. Aquaculture systems have been found to contribute to provisioning, regulating, and cultural ecosystem services (TEEB 2010; Gentry et al. 2020), notably regulating services by nutrient removal from areas suffering from nutrient pollution and loop closure of phosphorus (Thomas et al. 2021; Sinha et al. 2022), and biodiversity enhancements (Theuerkauf et al. 2021). While marine fisheries have stagnated since the 1990s, aquaculture has been rapidly growing (Naylor et al. 2021) to the extent that mariculture products now contribute significantly to food security and meeting protein demands globally (FAO 2020). However, the sustainability of such an expanding aquaculture sector has been called into question. Several negative environmental and social impacts have been documented, such as impact on wild fish stocks, degradation of habitats such as wetlands, genetic pollution, and poor conditions for workers (Primavera 2006; Young et al. 2019).

The rapid development of the aquaculture sector has put pressure on decision-makers across global and national levels to formalize governance for reduced negative environmental and social impacts, as well as to incentivize positive effects of the sector (Corner et al. 2020). Significant efforts have been put into governance for more sustainable aquaculture practices during the past 20 years (Naylor et al. 2021). One of these is the Ecosystem Approach to Aquaculture (EAA) which is based on three key principles: (i) Aquaculture development and management should take account of the full range of ecosystem functions and services, (ii) aquaculture should improve human wellbeing and equity, and (iii) aquaculture should be developed in the context of relevant sectors, policies, and goals (Brugère et al. 2019). Despite these efforts, legislation and policy are still lagging in many regions. This lag has created governance gaps or mismatches, often expressed in two governance pathways; the first being a lack of rules resulting in an unregulated industry with greater risks for both environmental and social implications (particularly in less developed countries), and the second being a situation with overlapping and poorly adapted regulations that constrain the development of the industry (mainly in developed countries) (Lebel et al. 2018). A key reason for the complexity of aquaculture governance is that aquaculture-related activities cover a range of different regulatory areas, notably environmental laws, marine spatial planning and use of space, animal health, and food safety (Hadjimichael et al. 2014).

Despite the expansion of aquaculture at a global scale, European aquaculture has stagnated over the past decades (Ertör and Ortega-Cerdà, 2017), particularly if only considering the EU member states and thus excluding the large Norwegian salmon industry. Approximately 55% of the current global aquaculture production is based on fed aquaculture of fish and crustaceans, while 44% is non-fed low trophic marine species (e.g., mollusks, seaweed, and ascidians) (FAO 2020). Low trophic mariculture (LTM) is also often referred to as the culture of extractive species, which draw sustenance from marine environments by absorbing dissolved nutrients or filtering out organic particles. LTM can, therefore, produce regulating ecosystem services (Gentry et al. 2020; Thomas et al. 2021) while also providing food and feed without the usual environmental and social pressures associated with fed aquaculture production (Gephart et al. 2021; Krause et al. 2022). While the EU’s share of global blue mussel production is approximately 25%, albeit declining (Innes et al. 2017), EU countries only contribute a fraction of the total production of seaweeds (FAO 2020). Simultaneously, the EU has developed strategies to support aquaculture and LTM in particular, notably the strategic guidelines for a more sustainable and competitive EU aquaculture (European Commission 2021b) and the strategy for blue growth (European Commission 2021a).

Hishamunda et al. (2012) argued that private sector entrepreneurs are the drivers of durable aquaculture. Consequently, an expansion of the aquaculture sector is hindered if these actors experience challenges that are not addressed in aquaculture governance. The selection of appropriate measures to promote sector activities and adapt governance requires a thorough understanding of the actors’ perceived hurdles for expansion. There are some studies exploring the causes of the limited expansion of the aquaculture industry in Europe. Commonly explored causes are governance constraints (Young et al. 2019), spatial planning (Corner et al. 2020), and social acceptance (Whitmarsh, and Palmieri 2009; Hynes et al 2017; Thomas et al. 2018; Billing et al 2021). Despite the general knowledge that can be extracted from these examples, studies addressing LTM aquaculture specifically (especially more than one species at the time), as well as including voices from the private actors themselves, are scarce or completely lacking. It can be hypothesized that the challenges and barriers facing aquaculture practitioners of fed aquaculture may differ from those working with LTM due to the inherent differences in production. The differences between fed and non-fed aquaculture may have implications for social acceptance, for licensing pathways and in relation to the Water Framework Directive (Directive, 2000/60/EC).

Therefore, this study combines LTM governance analysis and voices from the LTM private entrepreneurs, to understand how governance may affect development of the sector, focusing on the first step of the value chain, i.e., cultivation and harvest of low trophic species. As a case study, we use the LTM sector in Sweden, on the West Coast bordering the North Sea (Skagerrak), which is small and has only experienced nominal growth over the past decade. The paper adapts a two-folded approach by (i) analyzing how effective, equitable, responsive, and robust the current LTM governance is by studying literature and reports, and (ii) surveying the perception of LTM governance among the sector’s actors, i.e., private LTM business entrepreneurs. By analyzing LTM governance in combination with the perceptions of the LTM actors, lessons are learned, and barriers are identified for aquaculture governance and blue growth in Europe, notably for other countries facing similar stagnation in the LTM sector.

The paper is structured as follows. In “Materials and methods” section, the methods for mapping and analyzing LTM governance are presented, followed by the data collection methods used. In “Result” section, the outcomes of governance mapping and surveys of LTM business are presented. In “Analysis of LTM governance” section, we analyze what the result means in terms of how effective, equitable, responsive, and robust LTM governance is. In “Discussion and conclusions” section, it discusses our findings.

## Materials and methods

### Framework for understanding governance systems

**Fig. 1:**
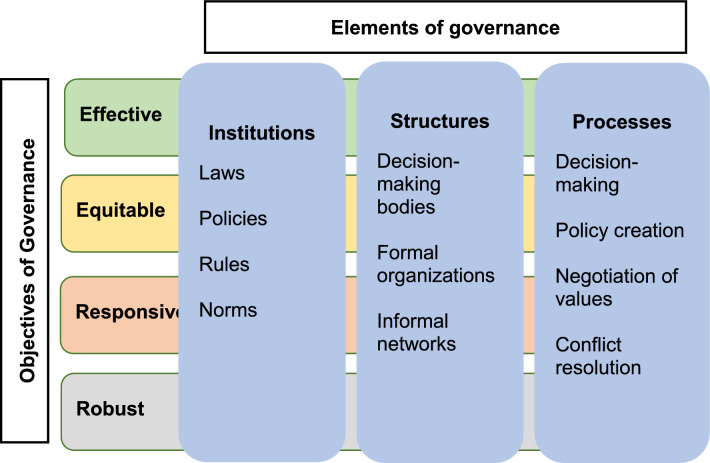
Framework for environmental governance adapted from Bennett and Satterfield (2018)

Dutra et al. (2019) summarized the core of understanding governance systems in three questions: (i) who is involved in making decisions, (ii) what influence and responsibilities do they have, and (iii) how are these responsibilities exercised. However, governance is often formed by a complex web of actors, decision-making structures and processes, rules and norms, formal and informal networks, and more features. Bennett and Satterfield (2018) suggest a practical framework for environmental governance to handle such complexity. The framework was developed through a comprehensive review of academic literature on environmental governance. The framework (Fig. 1) distinguishes three basic elements of governance: institutions, structures, and processes. *Institutions* are related to legislation, policies, norms, and other rules; *structures* are the decision-making bodies as well as formal organizations and informal networks; while *processes* are how decision-making works, such as policy creation and negotiation of values. Further, the framework presents four objectives of governance: effective, equitable, responsive, and robust. For each objective, the framework presents a set of defining attributes: *Effective* is related to attributes such as coordination, direction, capacity, and efficiency; *equitable* relates to recognition, participation, and fairness; *responsive* relates to the governance’s possibility to adapt and learn, be flexible, and innovative; and finally, *robust* can be described as how legitimate, connected, and nested governance is (Bennett and Satterfield 2018). Therefore, this framework offers a theory-based, but also practical, way of studying environmental governance and enables a structured way of mapping governance elements and analyzing the governance objectives.

### Data collection

The mapping of governance elements of the LTM sector focused on the licensing process for establishing and sustaining LTM activities that exist in Sweden today: farming of mussels (*Mytilus edulis*), oysters (*Ostrea edulis*)*,* seaweed (*Saccharina latissima*), and ascidians (*Ciona intestinalis*) and harvest of wild native oysters (*Ostrea edulis*) and pacific oysters (*Magallana*/*Crassostrea gigas*). In accordance with Bennett and Satterfield (2018), this included the identification of institutions (legislation, policies, and rules), structures (agencies involved in decision-making and relevant formal organizations such as producer organizations), and processes (decision-making, law making, and fora for trade-offs, negotiations of values, and conflict-solving). These elements were identified using the web-based platform for Swedish Aquaculture (Svenskt vattenbruk 2023) that has a comprehensive summary of the responsible and related authorities and legislation that concern aquaculture governance, and has been used for the governance mapping. The Swedish Board of Agriculture (SBA) is responsible for the platform. For further details on institutions and structures, as well as processes the study used literature reviews of academic literature as well as reports from national agencies, information from organizations related to LTM, and policy documents such as national policies. For an improved understanding of the licensing processes, the study used archived processes relating to permissions to operate for different types of LTM activities, by studying court cases. These were collected from the Land and Environmental Court of Appeal, at the Svea Court of Appeal in 2020. Even though the court cases are relatively old (2009–2013), they are often referred to regarding shoreline protection and licensing process for aquaculture (see, e.g., SBA 2020; Bet. 2022:18). A summary of the court cases can be found at a web-based platform of shoreline protection court cases, run by the Swedish County Administrative Boards (Strandskyddsdomar 2023).

To explore the perceptions of LTM governance in Sweden from a sector perspective, a stakeholder mapping was performed. As the sector is rather limited in size, the sampling strategy aimed to capture as many actors as possible. LTM businesses (private sector entrepreneurs) were identified using networks such as producer organizations and relevant reference groups related to the LTM sector. The mapping was delimited to the Swedish West coast, where 25 businesses were identified related to the first step in the value chain (operating in 2018). A web-based questionnaire was developed and distributed to the identified actors (businesses) in March 2019. These surveys were designed to get LTM actors’ perspective on factors affecting opportunities for the sector, such as values of the sector, perceived hurdles, necessary changes, and responsible actors for change. To increase the possibility of getting a good response rate, the questionnaire was limited to a few questions, including both closed- and open-ended questions (Appendix S1). The stakeholder questionnaire responses were anonymized and stored on a secure server. Sensitive personal data and personal identification data were neither collected nor stored and GDPR policies were adhered to. Recognizing that respondents may be possible to identify even when made anonymous, views, and opinions that may be traced back to individuals were not disseminated. Twelve of the 25 businesses answered the surveys, which means that the result cannot be interpreted as representing the entire LTM sector in Sweden. However, it can indicate how several LTM actors perceive the sector’s values and its hurdles.

## Results

Firstly, a brief introduction to the Swedish LTM sector is presented in “The Swedish low trophic mariculture sector” section. In “Governance elements” section, the result from the governance mapping is presented following the three elements of governance in the governance framework. The actors’ perception of the governance system is subsequently presented in “LTM actors’ perceptions” section.

### The Swedish low trophic mariculture sector

The LTM sector in Sweden is currently limited to the West Coast, bordering to the North Sea (Skagerrak). We found that currently, the LTM sector includes farming and harvesting of mussels, wild oysters, seaweed, and ascidians. In total, there were 25 businesses identified (as operating in 2018), using data from producer organizations, and knowledge from the researchers involved in the project (authors). Most businesses were small-scale entrepreneurs (under 10 employees) or family businesses, covering also other steps of the value chain such as retail or related activities such as tourism and gastronomy. Most businesses were working with oysters, followed by mussels. A few businesses worked with seaweed and even fewer with ascidians. Several businesses worked with more than one species. Cultivation of seaweeds and ascidians started off as research projects that have expanded to commercial activities, while oyster activities have been mainly driven by small-scale entrepreneurs, often as a part of other activities such as gastronomic tourism and/or mussel farming. Blue mussel farming was initiated in the 1970s and is the most widely produced species, but is, as in other parts of Europe, also in decline. The approximate amount of harvested biomass per year in the period between 2018 and 2020 in fresh weight was 1970 tons/year (mussels), 300 tons/year (ascidians), 49 tons/year (seaweed), and 9 tons/year (oysters). Consequently, the Swedish LTM sector is small but diverse (Sinha et al. 2022).

### Governance elements

#### Institutions

The most important laws affecting LTM activities identified in the study were the Fisheries Act (SFS, 1993:787) and the Environmental Code (SFS, 1998:808), regulating the LTM licensing processes. The cultivation of mussels and oysters is mainly regulated in the Fisheries Act, focusing on the licensing procedures. The cultivation of seaweed and ascidians, on the other hand, is regulated in the Environmental Code. This is because mussel farming, together with crayfish and fish farming, has an exemption from a regulation regarding permission for aquatic activities (in Swedish: *vattenverksamhet*) according to 11 c. 11§ in the Environmental Code (SFS, 1998:808). The exemption does not mention seaweed or ascidians, which means that licenses for culturing these species need to be preceded by an environmental impact assessment (EIA). The Environmental Code is also relevant for location for cultivations and precautionary measures (Kyrönviita 2022).

The Food Act (SFS, 2006:804) regulates food safety perspectives and harvest permissions, and is consequently (due to the study’s focus on the licensing process) less relevant for this study. The regulation, however, infers a demand of regular sample collection during the grow-out phase. There are different legislations that apply for the disposal and use of common water (SFS 1950:595) and privately own waters (SFS 1998:812). Further, for privately owned water, a permission from the owner is needed. In waters near the shoreline, LTM actors must also apply for an exemption from the shoreline protection according to the Environmental Code (SFS, 1998:808), regardless of whether the LTM actor owns the water or not. The shoreline protection generally applies to all shorelines in inland and coastal waters and is normally 100 m up on land and out in the water (totaling 200 m) but can be extended to 300 m (totaling 600 m). According to the 7 c. 16§ (SFS, 1998:808), facilities for the fishery, forestry, agriculture, and reindeer husbandry are exempted from shoreline protection. In praxis, these industries are called “food and natural resource dependent industries” (in Swedish: *areella näringar*). For permanent or temporary LTM facilities in the water, there are also rules for maritime safety regulating the use of navigation marks and updating maps for navigation, according to the regulation on maritime traffic (SFS 1986:300).

Regarding oysters, there is a difference between farming and harvesting the native *Ostrea edulis* oysters and the invasive pacific oyster *Magallana*/*Crassostrea gigas.* In the Fisheries Act, “oysters” is a resource exclusively belonging to the owner of the water, which means all picking of oysters needs to be approved by the property owner. However, “oysters” is not defined on a species level, leaving an unclear role of the law on pacific oysters. In Sweden, pacific oysters is defined as an invasive species which means that it is incorporated into regulations for invasive species in the Environmental Code and other national regulations. In contrast with other European countries, aquaculture of pacific oysters is not allowed in Sweden (Mortensen et al. 2019).

According to the final report of a simplification of aquaculture regulation processes (SBA 2020), the definition of “aquaculture” varies across institutions in Sweden. Consequently, the legislations and regulations that are applied to aquaculture in general do not harmonize, neither with respect to domestic legislations nor between domestic and EU legislations, such as article 4 paragraph 25 in the EU regulation on Common Fisheries Policy (Regulation 1380/2013). In some of these cases, “fisheries” also include aquaculture, whereas it is unclear in other cases.

In Sweden, the General Recommendations (Swedish: *Allmänna råd*) is a part of the regulatory hierarchy and can offer help on how to interpret and fulfill legislations or regulations. The latest such guidance on aquaculture from national authorities was developed by the Swedish Environmental Protection Agency in 1993 and only covered fish farms. This guidance was repealed in 2019, yet a new updated comprehensive guidance is still lacking (Kyrönviita 2022, p. 125). The responsible national authority for guidance today is the Swedish Agency for Marine and Water Management (SwAM).

From a national policy perspective, the strategy for Swedish fishery and aquaculture (SBA and SwAM 2021), a revision of this previous strategy for aquaculture 2012–2020, but also the Swedish Food Strategy (2017), are important. Both strategies are coupled with action plans, aimed to clarify and implement the strategies.

#### Structures

The mapping of governance structures identified ten national authorities as related to the governance of the LTM sector in Sweden (Appendix S2). Two of the most influential national authorities were the Swedish Board of Agriculture (SBA) and the Swedish Agency for Marine and Water Management (SwAM). SBA has the overall assignment to promote a sustainable aquaculture in Sweden. SBA is also responsible for registers relevant to LTM and has access to minor financial support (< 500 000 euros/year) related to the food strategy to support projects aligned with the action plan for aquaculture. SwAM has the overall responsibility for coastal and marine management, which includes regulatory guidance of aquaculture, responsibility for the Fisheries Act, and is responsible for the management of aquatic invasive species. Beyond SBA and SwAM, a range of other national decision-making bodies affect the LTM sector directly or indirectly, such as the Swedish Food Agency; the Swedish Transport Agency; the National Veterinary Institute; the Environmental Protection Agency; the Swedish Agency for Growth; the Swedish Maritime Agency; the Legal, Financial and Administrative Services Agency; and the Coast Guard.

On a regional level, the County Administrative Boards (21 in Sweden) have a key position for the aquaculture licensing procedures and decisions (Kyrönviita 2022), including LTM activities. They are state agencies based on the geographical and administrative level of county. Consequently, their responsibilities are not delimited to one specific topic, such as for national authorities like SBA or SwAM. The County Administrative Boards are responsible for permissions, registration, and licenses associated to a range of procedures (Appendix S2) for all types of LTM activities. For instance, they evaluate the environmental impact assessments for seaweed or ascidians cultures according to the Environmental Code, approve licenses for mussel farms and grant translocation permits based on the Fisheries Act, and issue exemption from shoreline protection for LTM culture. They also implement monitoring and control of LTM activities.

On a local level, the municipalities (290 in Sweden) are the local administrative units, with a politically elected municipal council. Municipalities are formally and exclusively responsible for the planning of land and water use (out to the territorial sea) according to the Planning and Building Act (SFS, 2010:900). They can appoint suitable zones for aquaculture in their comprehensive plans; however, these plans are not legally binding (Koglin and Pettersson 2017), and only a few municipalities have included zoning for aquaculture (Kyrönviita 2022, p. 232). Municipalities can also approve smaller cultivation sites.

In terms of formal producer organizations, there is an ongoing process of restructuring among the organizations related to LTM in Sweden. A former producer organization for shellfish was restructured to include other types of LTM species, such as algae and ascidians. A new organization, similarly covering LTM businesses, has also been established, but it is not yet classified as a producer organization by the SBA.

#### Processes

The mapping of governance processes focuses on licensing processes. Within these processes, important features from Bennett and Satterfield (2018), such as negotiation of values, conflict resolution, and application of policy, are also included.

For cultivation of LTM species, the businesses must apply for both cultivation license and exemption from shoreline protection, since aquaculture does not have the same exemptions from shoreline protection as other food and natural resource-dependent industries. The duration of the permits varies, both between different types of permits and for the same type of permit, often ranging from 3 to 10 years (SBA 2020). Moreover, permit processes may be lengthy, ranging from a few months to years.

To further understand how licensing processes for LTM businesses are affected by the fact that aquaculture is not exempt from shoreline protection in the Environmental Code, court cases of LTM activities’ exemption from shoreline protection on the Swedish West Coast were analyzed. In two of the cases (MÖD 2010-08-24, no. M 4240-09 and MÖD 2013-06-04, no. M 8456-12), a local nature conservation organization appealed the County Board’s decision for approving exemption from shoreline protection for LTM cultivation sites. The Land and Environmental Court approved the appeals from the nature conservation organization. The LTM business also appealed the Land and Environmental Court’s decisions to a higher jurisdictional level. The Land and Environmental Court of Appeal disapproved the appeals from the local nature conservation organization, based on the positive effects of LTM cultivation and the minor impacts on outdoor life (basis of the appeals). The third court case (MÖD 2009-05-12, no. M 1225-08) concerns exemption from shoreline protection for construction of facilities at the shore for oyster harvest. Also this court case was finally taken to the Land and Environmental Court of Appeal that determined—that an exemption from shoreline protection is not needed, since harvest of wild oysters is seen as fishery. These court cases illustrate how lengthy and costly licensing processes for LTM businesses could be, and also that harvesting and cultivation of LTM species are treated differently concerning the exemption from shoreline protection.

Regarding law making processes, there are several suggestions for revisions of legislation affecting LTM activities. During 2019–2020, the Swedish Board of Agriculture (SBA) was appointed the task by the Swedish government to review the possibilities of simplifying the licensing process for aquaculture (SBA 2020). The goal of the process was to identify major hurdles and inaccuracies in Swedish legislation for aquaculture and make suggestions on revisions. A working group including a range of actors (sector, NGOs, academia, and authorities) was recruited to provide specific expertise from different perspectives, which enabled the LTM sector to be active in the process and provide concrete input based on their experiences of barriers in the licensing procedure.

Regarding policy formation, the strategy for Swedish fishery and aquaculture (SBA and SwAM 2021) and the Food Strategy are important policy documents related to the LTM sector in Sweden. The first strategy illustrates a good example of where actors in LTM aquaculture were included in policy formation as the strategy is a result of a process involving relevant stakeholders from national to local authorities, producer organizations, researchers, and actors from the involved sectors. In contrast, the Food Strategy was developed without significant involvement of LTM aquaculture representatives.

Negotiation of values and fora for conflict-solving are important features in governance processes (Bennett and Satterfield 2018). Our study has not identified a systematic and transparent approach to conflict-solving or negotiation of values in relation to the licensing process.

### LTM actors’ perceptions

The questionnaire (Appendix S1) included questions regarding (i) the respondents’ view of values related to a viable LTM sector in Sweden, (ii) the main perceived hurdles for the achieving this, (iii) the changes needed to benefit the respondents’ businesses, and (iv) which actors are key for these changes and the development of the LTM sector in Sweden.

The dominant theme for (i) values of the LTM sector raised by respondents related to societal benefits to the coastal community. Respondents brought up increasing unemployment as a result of decreasing fishery, and thus, an area where the LTM sector has a potential of reducing this risk. Also, creating opportunities for small businesses and the local population to make a living was raised as potential benefits. The second most common theme was environmental benefits, such as improving water quality, uptake of nutrients, and improved marine environment. Three other themes were mentioned by more than one respondent: food production or food safety; events and local tourism; and increased knowledge and awareness of the sea.

Regarding (ii) main hurdles, all respondents filled in “legislation/regulations” as a hurdle. Three other alternatives were also prominent: “spatial conflict of interests,” “economic profitability” (i.e., financial profitability), and “knowledge gaps.” Only one respondent filled in “lack of demand” as a hurdle. In an open-ended question, respondents elaborated in more detail about these hurdles. The themes that concerned governance issues were thematized according to the elements of governance (Appendix S3). “Complex, complicated and counterproductive legislation” was the most common theme that respondents’ raised. Further, several respondents perceived a scepticism from decision-making bodies/authorities. Regarding decision-making and processes, respondents mentioned difficulties to get permissions, many appeals of the permissions, and a low degree of participation in, or influence on, decision-making from the actor in the LTM sector. “The spatial conflict of interest” was not clearly explained by respondents; however, the rules of shoreline protection were mentioned by several respondents as complicated, which could be related to conflict between spatial use for LTM activities versus other uses. “Economic profitability” was somehow elaborated such as expensive fees, difficulty to get profitability when permissions are difficult to get, and a lack of financial resources to build necessary infrastructure.

Regarding (iii) necessary changes, most respondents answered legislation, i.e., a simplification of legislation. However, several respondents also raised the attitudes from the authorities again and suggested a new and more positive approach to the LTM sector and its actors, including the possibility to participate and influence development. Several respondents also mentioned collaboration, such as the sector needs to cooperate, all different actors are important for change, and common networks. Related to this issue is the last theme (iv): on actors that are key for this change. The most common answers were authorities and policy makers, but many respondents also filled in the LTM actors (i.e., themselves and other private businesses actors in the sector). Nature conservation NGOs were also mentioned as a group making many of the appeals for the permissions.

In summary, we found that legislation was the most common theme of hurdles identified by the respondents. Respondents mainly described the existing legislation as complicated, but also unfair and counterproductive, where the legislation on shoreline protection was the most mentioned issue. Regarding structures, the most common theme entailed the attitude from authorities. Several respondents experienced a negative attitude from authorities and emphasized the need of changing general attitudes and perceptions of the sector toward a solution-oriented and developmental approach. Complicated, difficult, and expensive licensing processes were identified as a common theme regarding processes, but also appeals from NGOs were mentioned as a prominent hurdle. Other themes that appeared relating to several governance elements were the environmental benefits from LTM activities, a need to enable the sectors’ potential of producing protein-rich food and other societal benefits, and to strive for better collaboration within the sector and between the sector and other concerned actors.

## Analysis of LTM governance

We identified several circumstances in the governance mapping in “Governance elements” section and from the LTM actors’ perspective in “LTM actors’ perceptions” section which could affect the opportunities for the sector to be vital and potentially expand. The two data collections regarding governance are merged, and the main results are presented in Table 1, indicating potential hurdles mainly for institutions and processes. Clearly, current legislation is not adapted to new species such as seaweed and ascidians, and there are several issues on definitions regarding aquaculture and in particularly LTM that make interpretation of the legislation further complicated. Licensing processes are lengthy and complicated, and are in some cases worsen due to appeals regarding use of water areas within the shoreline protection. Above all, the study could not identify a proper arena or systematic mechanisms for resolving conflicts.

**Table 1:** Major characteristics of elements of governance for LTM in Sweden

Institutions	Structures	Processes
Legislation is not adapted to “new” species (such as seaweed and ascidians)	10 national authorities related to decision-making	Licensing process is complex, lengthy, and costly, perceived by LTM actors as an obstacle for expansion. Duration of different permissions is not aligned
Inconsistent definition of aquaculture in domestic legislation—creates challenges in interpretation of legislation	The County Administrative Boards (regions) are key actors for the licenses process	Law making processes—lengthy process, different suggestions on revisions of legislation has not been implemented
Lack of a comprehensive and updated guidance for how to interpret legislation for aquaculture in general	Municipalities (local authorities) have limited impact on licensing processes	Policy creation—participatory approach for the most important national strategy and action plans
A new national strategy for fishery and aquaculture was published 2021	Two producer organizations	Appeals from local organizations on exemptions from shoreline protection
LTM actors perceive legislation as complex and unpredictable	LTM actors experience a skepticism from authorities and request a solution and development-oriented approach	Lack of apparent arena or systematic approach for negotiation of values and conflict-solving for locations of LTM (licensing process)
Shoreline protection is particularly difficult and relates to further definitions issues (“*areell näring”)*		
Unclarities regarding legislation on oysters *gigas*		

In this section, we analyze the findings from the four LTM governance objectives: effective, responsive, equitable, and robust using this governance mapping and the voices from the sectors.

### Effective

Bennett and Satterfield (2018) explicate *effective* environmental governance, in the applied framework, with the attributes of direction, coordination, capacity, informed, accountable, and efficiency. There are several features from the study which do not align with these attributes. One example is the lack of a clear collective vision which could have shaped policies and guided action for the LTM sector in a clear direction. There is a collective vision in national strategy documents; however, the LTM actors in this study do not experience that this vision spreads to real implementation. This creates a gap between the vision and the implementation of the visions.

Further, several obstacles to effective governance for Swedish LTM seem to be related to ambiguous definitions. There are different definitions of “aquaculture” in different Swedish institutions, and they are not aligned with the common definition from the EU level. Further, “food and natural resource dependent industry” is a commonly used concept in guidance documents and policies related to shoreline protection (e.g., SOU 2015:108; SEPA, 2024; SOU 2023:103). Yet, the concept has no clear definition in a legal context. The legislation on exemption from shoreline protection in the Environmental Code clarifies which industries the exemption applies to, and in that list “aquaculture” is not included. Since this clearly hinders the aquaculture sector in Sweden, a Government Official Report on “Food and natural resource-dependent industries by the water” (In Swedish: *Areella näringar vid vatten*) suggested that “aquaculture” is incorporated in 7 c. 16§ in the Environmental Code (SOU 2023:103). If this suggestion is realized, the definition of aquaculture needs to be clarified and aligned with EU. Impact analyses on this suggestion are conducted in both the report of simplification of aquaculture regulation process (SBA 2020) and in the new governmental report mentioned above (SOU 2023:103).

The conflict of interest related to LTM activities and the use of the shoreline revealed in this study, is a severe obstacle, resulting in lengthy licensing processes, administrative burden, pressure on the decision-making processes, and increasing conflicts between different activities. It does not align with Bennett and Satterfield’s (2018) idealized output of *efficient*: “Maximizes the productivity of management actions, while minimizing the wasteful use of available resources.” Within *capacity*, the framework also includes mechanisms to resolve conflicts. The findings indicate a lack of a proper arena for conflict-solving, which can further aggravate inefficiency regarding the conflict of interest of the shoreline usage. *Coordination* of roles, functions, and mandates among different agencies and organizations is also an attribute to effective in the framework. Considering the complexity of LTM activities, covering a range of regulatory areas including different legislation and responsible authorities, coordination could be argued to be central for effective LTM governance. The study identified ten national authorities, as well as regional and local authorities to be a part of the LTM governance structure. Yet, there is no coordinating body or an agency having this function today.

### Equitable

Some of the respondents’ perception of the LTM governance in Sweden relates to the “equitable” objective and the attributes of recognition, participation, fair, and just. The respondents emphasize the need of a more positive attitude and *recognition* from authorities, and a more participatory and solution-oriented approach to development of the sector (increased *participation*). Further, Bennett and Satterfield (2018)* argues that* the governance system should “ensure a fair balance of costs and benefits accrue to different groups”). If different LTM businesses and other marine industries (such as fishery) are seen as different groups in this regard, the result indicates several issues that are unfair in governance: the differences in primary legislation for LTM business (including the EIA process for ascidians and macroalgae) and the fact that aquaculture (and thus LTM) is not today included in “food and natural resource dependent industries” as discussed above. This connects to one of the obstacles identified in the study: the exemption from shoreline protection. If LTM (and aquaculture in general) was considered equal to other food production industries such as agriculture, fisheries, and reindeer husbandry, it could create improved conditions for the sector. Concrete examples could be exemptions in crucial legislations as well as enabling earmarked economic support for LTM businesses (SBA 2020). Both these issues were explored in the government assignment led by SBA; however, further investigations were suggested. From another perspective, one could argue that the legislation on shoreline protection is *just* according to Bennet and Satterfield’s (2018) definition, by enabling that all groups have access to justice. This is an important legislation for the public’s access and right to the shoreline, for all groups in society. However, it creates lengthy and costly processes for LTM businesses and also reduced trust between different users of the shoreline.

### Responsive

Responsive is characterized by the attributes of learning, anticipatory, adaptive, innovative, and flexible. The most obvious deviation from the objective from this study relates to *adaptive*, for which Bennett and Satterfield (2018) summarize the idealized output as: “Ensures that management plans and actions are being actively adapted to reflect changing social-ecological contexts and new knowledge.” Even though the benefits of macroalgae and ascidian farming are well known (Hasselström et al. 2018), their cultivation is governed by legislation that is more focused on damage control compared to the legislation surrounding the cultivation of mussels and oysters. Suggestions on simplification of legislation were initiated already in 2011 but suggested solutions and adaptions are yet not decided.

Respondents in the study also emphasize hurdles related to the “responsive” objective and highlight in this way some challenges to Swedish governance related to *learning*, *adaptiveness*, and enabling *innovation*. Here, it is important to relate back to coordination and the lack of platforms for negotiation of values and conflict-solving, not only when producing strategies but in real implementation and licensing processes affecting the LTM actors’ every-day life and possibilities to operate in the sector. *Flexibility* is also a term related to “responsive,” which Bennett and Satterfield (2018) mean is related to the need of policies that recognize local realities, where the policies are applied. The relatively small impact that local authorities, i.e., municipalities, have in Swedish LTM governance does not amplify this objective.

### Robust

Regarding “robust,” there are also clear inconsistencies with the applied framework in this study and the findings. The objective and its attributes (legitimate, connected, nested, and polycentric) are closely related to the performance of institutions. Complex and inconsistent legislation was one of the main results from the survey in the study and the governance mapping. According to the framework, *legitimate* governance is characterized by “A collective vision shapes policies and guides actions at all scales” (Bennett and Satterfield 2018). This is not aligned with the result from this study. One of the most important findings are that despite mussels, oysters, ascidians, and macroalgae having similar functions in the ecosystem and being cultured using similar techniques, they are primarily regulated by two different legislations. Ascidians and macroalgae cultivations must be preceded by an environmental impact assessment (EIA) which is an expensive and lengthy process involving obligatory processes of public consultation. Another potential challenge for legitimacy is the lack of updated guidance for how to make decisions based on existing legislation. The results in this study indicated inconsistencies in existing legislation and definitions that affect the results of the licensing processes. *Connected* is, as for the “effective” objective, also linked to the “robust” objective. The lack of coordination, and the existence of a coordinating body, or bridging agency, is mainly elaborated in 4.1.

## Discussion and conclusions

This paper aimed to understand how governance is affecting opportunities for the Swedish low trophic mariculture (LTM) sector. The paper explored LTM governance and hurdles perceived by the LTM sector’s entrepreneurs. We found that the two approaches of data collection methods created an improved understanding for what is characterizing LTM governance in Sweden and its role in expansion of the LTM sector. However, only about half of the Swedish businesses answered the survey, which should be taken into account when analyzing the results from the survey. Future studies would gain from a broader representation of the LTM businesses.

Following Bennett and Satterfield’s (2018) framework, the result indicated that the LTM governance for several reasons is not aligning with the framework’s objectives: effective, equitable, responsive, and robust. This is creating costly and unequal conditions, as well as ambiguous legislation and practice of licensing processes for different LTM businesses. Some features, such as legislation and regulations, takes a lot of time, resources, and considerations to change. Hasty and unconsidered changes of legislation would not align with the objective of robust governance. However, the study also identified a lack of coordination or a bridging functioning agency for authorities, organizations, and LTM entrepreneurs, as well as a lack of systematic approach to conflict-solving. Actions to fill these gaps would probably have a positive impact on LTM governance and could reduce some of the negative implications of the complex institutional landscape, and improve the licensing process. Studies from other countries also point out a lack of coordination between regulatory authorities as a barrier for expansion (Corner et al. 2020; Renwick 2018). Future studies exploring different solutions for coordinating roles and bridging organization for LTM in different countries and governance settings would be valuable.

The role of governance for aquaculture development should not be underestimated (Hishamunda et al. 2012). Governance is crucial for reducing negative social and economic effects, but equally important to strengthen positive effects of a sector. The LTM sector has the potential of producing several societal benefits, such as protein-rich food, ecosystem services, and employment (Krause et al. 2020; Naylor et al. 2021; Thomas et al. 2021). If LTM businesses produce commercially valuable products, this means that they are achieving genuine blue growth, i.e., producing both private and public benefits (Hasselström and Gröndahl 2021). The LTM sector in Sweden is characterized by small-scale entrepreneurs covering several steps of the value chain, and being closely related to tourism and gastronomy. However, the sector experiences severe governance-related hurdles in operating and expanding LTM activities. Some of the hurdles identified in the study, particularly those related to the complex licensing process, are reported in many studies (Renwick 2018; Young et al. 2019; Corner et al. 2020) and recognized and addressed in EU strategies for aquaculture. Commonly suggested solutions to reduce the administrative burden and shorten licensing processes are to streamline legislation and aquaculture guidance, setting up an aquaculture entity that coordinates relevant authorities, and creating “one-stop-shop” systems for licensing procedures (European Commission, 2021b). Notably, many regulations that LTM actors perceive as obstacles are domestic legislation, such as shoreline protection, and not incorporations of EU directives into Swedish law. This may be due to aquaculture, in general, not being regulated by the EU in the same way as fisheries. Aquaculture is, therefore, primarily dependent on domestic legislation (Stead 2019).

Yet, some of the hurdles identified in the study are more connected to the unclear role of LTM in Swedish governance, including the precautionary attitudes and lack of recognition LTM actors experience from regulating authorities. Many LTM activities are regulated through environmental legislation, which mainly regulates toward avoiding or reducing potential disturbances to the coastal and water environment. However, this same legislation is not intended to account for positive environmental effects of LTM, which, therefore, are not incorporated in decisions and licensing processes. This is compounded by the lack of arenas or systematic approaches for negotiation of values or trade-offs. This type of nested social–ecological system must be governed with flexibility and participatory approaches, since the LTM sector is dependent on its operating actors. Challenges perceived by the sector’s actors thus need to be addressed in decision-making. If aquaculture, and particularly LTM activities, is supposed to contribute to the genuine blue growth development in Europe, and elsewhere, societal values and participatory approaches must be considered in LTM governance and its implementation, not only in strategies.

## Supplementary Information

Below is the link to the electronic supplementary material.

Supplementary file1 (PDF 780 KB)
