# Supplementary material for: Governance hurdles for expansion of low trophic mariculture production in Sweden
Source: Ambio. 2024 May 6;53(10):1466–78. doi: 10.1007/s13280-024-02033-4 (PMC11383906; doi:10.1007/s13280-024-02033-4)
Supplement: Supplementary file 1 — Supplementary file1 (PDF 780 KB) [file 13280_2024_2033_MOESM1_ESM.pdf]

Governance hurdles for expansion of low trophic mariculture production in Sweden

Franzén, F.[1], Strand, Å.[2], Stadmark, J.[3], Ingmansson, I.[1], Thomas, J-B. E.[4*] Söderqvist, T.[5,6] Sinha, R..[4] Gröndahl, F..[4] Hasselström, L.[4]

[1]Tyrens AB, Folkungagatan 44, SE-118 86 Stockholm

[2]IVL Svenska Miljöinstitutet / IVL Swedish Environmental Research Institute, Kristineberg 566, 451 78 Fiskebäckskil, Sweden

[3]IVL Svenska Miljöinstitutet / IVL Swedish Environmental Research Institute, Box 530 21, 400 14 Gothenburg, Sweden

[4]KTH Royal Institute of Technology, Stockholm: Dept of Sustainable Development, Environmental Science and Engineering. Teknikringen 10B, SE-100 44 Stockholm, Sweden

[5]Anthesis Enveco AB, Barnhusgatan 4, SE-111 23 Stockholm, Sweden

[6]Holmboe & Skarp AB, Norr Källstavägen 9, SE-148 96 Sorunda, Sweden

*** Correspondence:**
Thomas, J-B. E.
jbthomas@kth.se

Ambio

Supplementary Information
This supplementary information has not been peer reviewed.

Title: **Governance hurdles for expansion of low trophic mariculture production in Sweden**

Appendix 1

The original list of questions is provided hereafter in Swedish, followed by a translation of the questions (undertaken by the authors) in English for this supplementary material.

Questionnaire in Swedish

1. Vilken eller vilka av följande typer av vattenbruk/arter är din verksamhet kopplad till?

Alternativ: Ostron, Alger, Musslor, andra extraktiva arter

2. Beskriv på vilket sätt din verksamhet/sysselsättning kopplar till de extraktiva vattenbruk du kryssat för i föregående fråga.

Fritextsvar

3. Könstillhörighet

Alternativ: Kvinna, Man, vill ej definiera

4. Vad ser du för värden med ett levande extraktivt vattenbruk på Västkusten? (T ex för lokalsamhällen, miljö, kulturella eller sociala värden). Ge exempel.

Fritextsvar

5. Inom vilket/vilka områden upplever du att det finns hinder för extraktiva vattenbruk?

a. Lagstiftning/regleringar
b. Målkonflikter med andra intresseområden om områdesanvändning
c. Ekonomisk lönsamhet
d. Bristande kunskap
e. Teknisk utrustning
f. Infrastruktur
g. Bristande efterfrågan

6. Motivering till svar på frågan ovan. Skriv gärna en förklaring till de hinder du upplever som störst/mest påtagliga från ditt perspektiv eller din verksamhet.

Fritextsvar

7. Vilka slags förändringar skulle underlätta för din verksamhet/ sysselsättning? (kan bara välja en)

a. Lagstiftning
b. Ekonomiskt stöd
c. Forskning/mer kunskap
d. Annat

8. Motivering till svar på fråga ovan. Skriv gärna en förklaring till de förändringar du upplever skulle göra störst nytta från ditt perspektiv eller verksamhet.

Fritextsvar

9. Vilka av följande aktörer tror du är centrala för att få till stånd utveckling av extraktivt vattenbruk?

a. Myndigheter /politiker
b. Intresseorganisationer
c. Konsumenter
d. Näringsidkare
e. Akademi

10. Motivering till svar på fråga ovan. Skriv en förklaring till de aktörer du upplever skulle göra störst nytta från ditt perspektiv eller verksamhet.

Fritextsvar

English translation of questionnaire

1. Which low trophic species are you involved with?

Options: Oysters, Seaweed, Mussels, other extractive species

2. Describe your business

Open question

3. Gender

Options: Female, Male, do not want to disclose

4. Which values do you see in a viable low trophic sector in Sweden? (E.g. for local communities, environment, cultural or social values). Give examples.

Open question

5. In which of the listed categories below do you perceive that there are hurdles for low trophic mariculture?
   a. *Legislation/regulations*
   b. *Spatial conflict of interests*
   c. *Economic profitability*
   d. *Knowledge gaps*
   e. *Technical equipment*
   f. *Infrastructure*
   g. *Lack of demand*

6. Motivation for your answer to the question above. Feel free to write about the key obstacles you perceive from your perspective or for your business.

Open question

7. What kind of changes would be beneficial from your perspective or for your business? (can only choose one)
   a. *Legislation*
   b. *Economic support*
   c. *Research/knowledge*
   d. *Other*

8. Motivation for your answer to the question above

Open question

9. Which of the following actors do you think are key to the development of extractive aquaculture?
   a. *Authorities/policy makers*
   b. *Interest groups*
   c. *Consumers*
   d. *Sector actors*
   e. *Researchers*

10. Motivation for your answer to the question above

Open question

Appendix 2. Citations from open ended questions in survey to LTM actors, sorted in the elements of governance. *Italic text* indicates what is needed in the future.

	Institutions	Structures	Processes
	"Legislation is adding obstacles by many regulations, proprietorship, hurdles for farming…"<br>"The legal issues are the major hurdle, proprietors, fishery legislation… and shoreline protection makes it hard to create necessary infrastructure on land"<br>"Shoreline protection with 300 meters is the worst… It should be equal too all, it is not that way today"<br>"Complicated legislation… new complicated regulations from the Swedish Maritime Agency for professionally register boat…"<br>"Why is it forbidden to cultivate *gigas* when it is impossible to hinder its spreading"<br>"Simplification would imply better profitability…"<br>*"Simple clear regulations which gives predictability and possibilities. Investors are interested, but not if it is too many risks"*<br>*"Proprietorship. Ownership to an invasive species. Change it and the possibilities for the sector will change"*<br>*"Legislation, totally horrible and counterproductive"*<br>*"Legislation needs to be changed"*<br>*"Why is aquaculture actors exempt from the fuel tax relieves that farmers and foresters have?"*<br>*"Economic support for the environmental benefits from mussel farming, legislation that enables the use of rest products from farming"*<br>*"Compensation for the environmental benefits… "*<br>*"The proprietorship of gigas"*	"Easy for fishery officials to reject applications based on environmental argumentation such as N2000, protected areas etc."<br>"Focus on development, instead of complicating things"<br>*"Authorities needs to be more solution oriented. The [LTM] sector needs to see its common interest… "*<br>*"A positive approach from local/regional and national authorities coupled to help and support"*<br>*"Sweden has very much to learn from other countries… in the case of the officials' attitude to development, particularly in the coastal zone."*<br>*"We experience a clear skepticism from authorities to start great aquaculture [LTM] projects, particularly when it comes to off-shore farms"*<br>*"Another more positive attitude for producing food"*<br>*"… the sector needs authorities that see the possibilities before the problems, which is not the case today"*	"Appeals from NGO's"<br>"Too much focus on control, no apparent interest in development"<br>"Expensive fees to start cultivation"<br>"Very difficult and demanding to get permission, [exemption from] shoreline protection must be applied for in short intervals involving fees every time."<br>*"Possibilities to establish a permanent farm if everything works out according to regulations. To avoid permission processes for cultivation and exemption from shoreline protection periodically"*<br>*"NGOs must stop appealing extractive aquaculture…"*<br>*"We who work in the sector have great knowledge and should have an influence in how the processes could be simpler and not so complicated"*<br>*"A clear and simple way [procedure] to start cultivation, it is as important as environmental monitoring of the activities"*<br>*"All actors are needed [in 'necessary changes'] but in different forms and steps. Only by cooperation can we develop"*

	"Simplification of regulations"		
